# Neural activity in adults with major depressive disorder differs from that in healthy individuals: A resting-state functional magnetic resonance imaging study

**DOI:** 10.3389/fpsyt.2022.1028518

**Published:** 2022-11-17

**Authors:** Xiaofang Hou, Bohui Mei, Fukun Wang, Hua Guo, Shilong Li, Gang Wu, Chen Zang, Bing Cao

**Affiliations:** ^1^Laboratory of Magnetic Resonance, Zhumadian Second People’s Hospital, Zhumadian, Henan, China; ^2^General Committee Office, Zhumadian Second People’s Hospital, Zhumadian, Henan, China; ^3^Committee Office, Zhumadian Second People’s Hospital, Zhumadian, Henan, China; ^4^Department of Medical, Zhumadian Second People’s Hospital, Zhumadian, Henan, China; ^5^Laboratory of Computed Tomography, Zhumadian Second People’s Hospital, Zhumadian, Henan, China; ^6^Key Laboratory of Cognition and Personality, Faculty of Psychology, Ministry of Education, Southwest University, Chongqing, China

**Keywords:** MRI, resting-state, major depressive disorder, brain, functional connectivity

## Abstract

**Objective:**

Currently, findings regarding resting-state functional magnetic resonance imaging studies of major depressive disorder (MDD) are inconsistent. In contrast to the previously used *a priori* seed-based functional connectivity analyses, this study employed whole-brain exploratory analyses and aimed to explore neural activity patterns in Chinese adults with MDD.

**Materials and methods:**

Specifically, this study examined the amplitude of low-frequency fluctuations within the whole brain and adopted a large-scale brain network template to explore the core dysfunctional brain regions in individuals with MDD.

**Results:**

Overall, 32 individuals with MDD and 32 healthy controls were evaluated. Compared to healthy controls, individuals with MDD showed more profound alterations in the amplitude of low-frequency fluctuations in the temporolimbic affective circuit (e.g., middle temporal gyrus and parahippocampus) and default mode network (e.g., precuneus and thalamus). Moreover, functional connectivity between the left mid-insula and parietal regions within the sensorimotor network was weaker in individuals with MDD than in healthy controls.

**Conclusion:**

In conclusion, the neural characteristics of MDD correspond to cognitive deficits in self-referential processing and emotional processing and are related to a risk of sensory disorders or psychomotor retardation. These findings present neural markers that may be used to identify MDD, contributing to clinical diagnosis.

## Introduction

Depression is a psychiatric disorder that clinically manifests as significant and persistent depressed mood and loss of pleasure and interest along with somatic and cognitive changes, and patients with severe cases have suicidal ideation (1, 2). In 2008, the World Health Organization declared major depressive disorder (MDD) to be the third leading cause of the global burden of disease, annually affecting approximately 350 million people worldwide, and it is projected to become the leading global disease by 2030 (3). MDD is more than just an affective disorder (4) and is often associated with reduced quality of life and psychosocial functioning (5). Patients with MDD often have cognitive deficits in executive functioning, decision making, and emotional processing (6). MDD is also a mental disorder (7) often associated with neural processing defects. Therefore, revealing the pathogenesis and causes of MDD from the perspective of neural activity can help in its identification and treatment.

Resting-state functional magnetic resonance imaging (fMRI) is often used to explore neural activity patterns in MDD, reflecting brain activation at rest (8). Mayberg et al. (9) initially proposed an influential neural model of MDD that states that activity in the limbic and paralimbic structures (e.g., hippocampus) that supports emotional processing can dominate the activity in the dorsal cognitive cortex (e.g., dorsolateral prefrontal cortex) (9). Hamilton et al. (7) advanced this theory by determining the directions of neural interactions between different brain structures using Granger causality analysis (7). They found that the limbic system in patients with MDD enhances excitatory activity in paralimbic structures and inhibits dorsal cortical structures. These abnormal patterns of effective connectivity underscore the corticolimbic inhibitory effects of the limbic system on the dorsal cortex, an important neural cause of depression. In addition to frontolimbic structures (10–15), the temporolimbic system is also considered as a crucial neural marker in MDD. For example, Wu et al. (16) found stronger activation within temporolimbic structures, including the anterior cingulate gyrus, insula, and parahippocampal gyrus, but weaker activity in most frontoparietal cognitive regions in patients with MDD compared to healthy controls (16). Analysis using the amplitude of low-frequency fluctuations (ALFF) and graph theory showed greater recruitment of bilateral temporolimbic regions and a reduction in local functional segregation in MDD (17). These results suggest that the corticolimbic structures associated with emotional regulation are neural markers for identifying and differentiating MDD (18–21).

Resting-state functional connectivity (FC) refers to the temporal coherence of the blood oxygen level-dependent signal between different regions, and this coherence indicates the neural communication patterns within or between brain networks (22). Previous studies have held different views regarding which brain networks are most associated with MDD (23, 24). Kaiser et al. (25) suggested that the inconsistent results may be due to the use of various methods (25). They performed a meta-analysis and demonstrated that patients with MDD displayed neural abnormalities in a set of areas involved in internally (e.g., default mode network) and externally (e.g. dorsal attention network) oriented attentional systems as well as emotion processing and regulation (e.g., frontoparietal network). Greicius et al. (26) highlighted the important role of the default mode network in MDD (26). Liu et al. (27) recently revealed dysfunctional activity in an extensive range of brain networks, including the default mode network, central executive network, limbic system, visual network, somatomotor network, ventral attention network, and dorsal attention network in patients with MDD (27). In general, MDD is characterized by altered connectivity in multiple networks involved in cognition and emotion processing.

The ALFF is an indicator of activity in resting-state fMRI that can be used to represent spontaneous brain activity (28, 29). ALFF is considered a reliable and sensitive measure in clinical population studies (30) and is a classic indicator used to detect neural activity fluctuations in patients with MDD (17, 31). In addition to the analysis of neural activity, FC analysis can examine the communication between neural networks and brain regions. Most previous studies employed seed-based voxel-wise FC analyses according to explicit hypotheses from other studies (27). Seed-based voxel-wise FC analysis is a whole-brain FC analysis that selects key brain regions identified in previous studies as seed points. Few studies have conducted exploratory FC analyses on large-scale templates. Dosenbach’s 160 regions of interest (ROIs) template is a weighted network derived from previous fMRI meta-analysis of cognitive tasks (32). It contains information about the FC strength and is considered the best template for building brain functional networks (33).

The present study aimed to investigate neural abnormalities in individuals with MDD using resting-state fMRI. Whole-brain ALFF and exploratory FC approaches were employed to explore altered brain activation and FC changes. ALFF and Dosenbach’s 160 atlas were used to explore the characteristics of spontaneous neural activity in patients with MDD. Thus, it was predicted that distinct ALFFs and FC were discovered in brain regions related to mood regulation and cognitive control of attention compared between patients with MDD and healthy controls. This study attempted to understand the altered neural activity in patients with MDD and to summarize the neural markers that can identify and diagnose clinical depressive symptoms.

## Materials and methods

### Ethical approval

This study was approved by the Medical Ethics Committee of Zhumadian Second People’s Hospital in Henan Province (Approval no. IRB-2020-006-02) and was conducted according to the principles of the Declaration of Helsinki. All participants provided written informed consent prior to participation.

### Participants

Thirty-two patients diagnosed with MDD at Zhumadian Second People’s Hospital in Henan Province were recruited. The exclusion criteria were as follows: (1) any history of neurological diseases, other physical diseases, or comorbidities of other disorders; (2) any other mental disorders, such as schizophrenia; (3) pregnancy or breastfeeding; and (4) head trauma resulting in loss of consciousness. As controls, 32 age- and sex-matched healthy participants were recruited. The healthy controls had no history of mental illness or serious physical illness and no family history of mental illness.

MDD was diagnosed according to the Diagnostic and Statistical Manual of Mental Disorders-Fifth Edition criteria. The Hamilton Depression scale-17 (HAMD-17) score threshold was set at ≥ 18. All patients were diagnosed by two professional and experienced psychiatrists.

### Data acquisition and processing

All MRI data were obtained using a 3-T Trio scanner. Resting-state fMRI images were acquired using a gradient echo-planar imaging sequence. The sequence parameters were as follows: repetition time, 2,000 ms; echo time, 30 ms; flip angle, 90°; thickness, 4 mm; slices, 33; and field of view, 220 mm × 220 mm. Image preprocessing included the following steps. The first 10 images of each participant were removed for signal stabilization. The remaining images were corrected for slice timing and head motion. The functional and T1 images were reoriented to the standard Montreal Neurological Institute (MNI) template. Each subject’s functional images were registered to their high-resolution T1-weighted anatomical images. T1 images was segmented into white matter (WM), gray matter and cerebral spinal fluid (CSF). The linear trend, Friston 24 head motion parameters and nuisance signals from WM and CSF were regressed out (34). Next, each image was spatially normalized to the standard MNI template and voxels were resampled to 1 × 1 × 1 mm^3^ and then smoothed with a 6-mm full width at half maximum Gaussian kernel. Finally, the obtained images were band-pass filtered (0.01–0.08 Hz) and linearly detrended.

### Amplitude of low-frequency fluctuations analysis

ALFF was calculated according to the method proposed by Zang et al. (35). The time series for each voxel were first transformed into the frequency domain, and the square root of the power spectrum was calculated for each voxel and averaged over the specified frequency range (0.01–0.08 Hz). The averaged square root was used as the ALFF (36). To examine group differences in spontaneous brain activity, a two-sample *t*-test was conducted on whole-brain ALFF maps of the two groups at the voxel level. The results were corrected for multiple comparisons using the generalized random forest method, with the threshold set at *P* < 0.001 at the voxel level and *P* < 0.05 at the cluster level. All analyses were performed using DPABI software (37).

### Functional connectivity analysis

Dondenbach’s template parcellated the brain into 160 functionally segregated ROIs. These 160 ROIs consisted of six functional networks: default, frontoparietal, cingulo-opercular, sensorimotor, occipital, and cerebellar networks (32). In the current study, the ROIs were delineated into 160 spheres with a radius of 5 mm according to the node coordinates of Dondenbach’s template. The blood oxygen level-dependent signals of each ROI were computed by averaging the signals of all voxels within each ROI. ROI-by-ROI Pearson correlation coefficients were calculated and a 160 × 160 FC matrix obtained for each participant. To explore group differences in FC, the resultant connectivity was compared between the two groups using a two-sample *t*-test. The results were held at a threshold based on a false discovery rate of *P* < 0.05.

## Results

The mean age of the MDD group was 34.72 ± 9.05 years, and 53.13% (15/32) of the patients were women. While no significant differences in age, sex, or body mass index were observed between the two groups (*P* > 0.05), years of education were significantly lower in the MDD group than in healthy controls (8.53 ± 3.33 vs. 12.43 ± 3.62, *p* < 0.0001). In the MDD group, the mean duration of illness was 3.31 ± 3.15 years, and the mean HAMD-17 total score was 32.40 ± 9.35. The participant characteristics are presented in Table 1.

**TABLE 1:** Basic information of the included participants.

Variable	MDD (*n* = 32)	Healthy controls (*n* = 32)	*P*-values
Age, year, Mean ± SD	34.72 ± 9.05	34.25 ± 9.71	0.842
Sex (female/male); n/%	17/15 (53.13/46.88)	18/14 (56.25/43.75)	0.802
Education, year, Mean ± SD	8.53 ± 3.33	12.43 ± 3.62	<0.0001
Duration of illness in years, Mean ± SD	3.31 ± 3.15	–	–
HAMD-17 total score, Mean ± SD	32.40 ± 9.35	–	–
BMI, kg/m^2^, Mean ± SD	22.44 ± 2.70	22.13 ± 3.95	0.715

**FIGURE 1:**
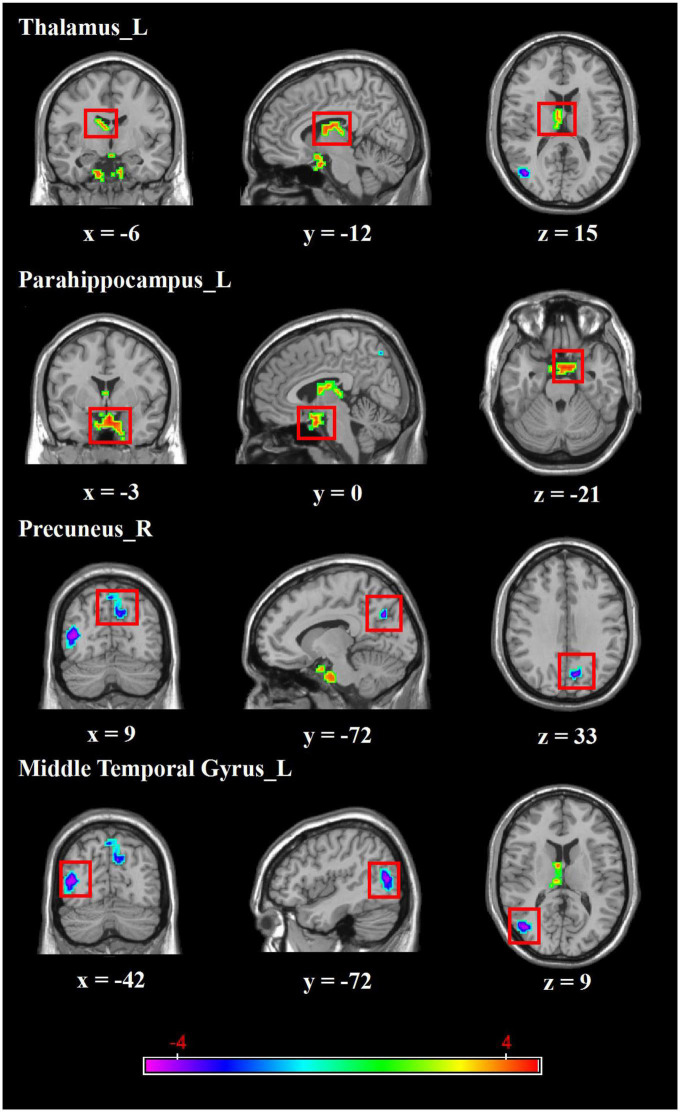
Brain regions with abnormal ALFF between MDD group and healthy controls. Red colors show the stronger ALFF in MDD group than that in healthy controls, and blue colors denote weaker ALFF in MDD group. Color bar represents the *T*-value (GRF correction, cluster-*P* < 0.05, voxel-*P* < 0.001). L, left, R, right.

Regarding group differences in ALFF (Table 2 and Figure 1), the MDD group showed higher ALFF in the left parahippocampus and left thalamus than the healthy control group. However, ALFF in the right precuneus and left middle temporal gyrus were lower in the MDD group than in the healthy controls.

**TABLE 2:** Brain regions where ALFFs were different between individuals with MDD and healthy controls.

Regions	Side	Peak MNI coordinates			Voxel size	*T*-value
		x	y	z		
**MDD > Healthy controls**						
Parahippocampus	L	−3	0	−21	180	4.728
Thalamus	L	−6	−12	15	70	4.4809
**MDD < Healthy controls**						
Precuneus	R	9	−72	33	46	−4.7022
Middle temporal gyrus	L	−42	−72	9	57	−4.4249

MNI, Montreal neurological institute; MDD, major depressive disorders; L, left; R, right.

**FIGURE 2:**
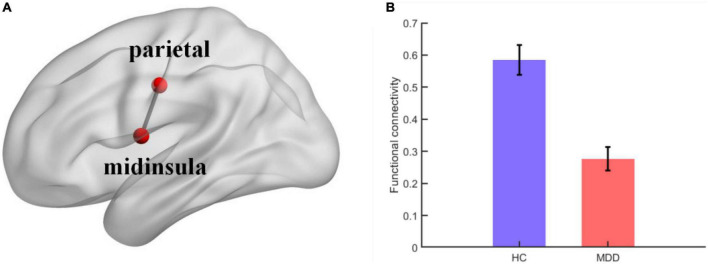
**(A)** Visual depiction of midinsula-parietal functional connectivity within sensorimotor network; **(B)** column chart depicting the midinsula-parietal FC differences between individuals with MDD and healthy controls. The error bar represents the within-subject standard errors of the mean.

FC analyses showed significant differences within the sensorimotor networks. Specifically, the FC between the left mid-insula (x = −42, y = −3, z = 11) and left parietal cortex (x = −47, y = −12, z = 36) was weaker in the MDD group than in the healthy control group (*t* = 5.6393, *p* < 0.001) (Figure 2).

We further conducted correlation analysis to assess the association between brain imaging indicators and clinical data. No significant correlations were found with a significance threshold of *P* = 0.05 (details not shown).

## Discussion

Given that resting-state fMRI studies of MDD have reported varied findings, the present study aimed to provide new evidence using new analyses. Using whole-brain resting-state fMRI and FC analyses, the current study found that, compared with healthy controls, patients with MDD showed stronger ALFF in the left parahippocampus and thalamus and weaker ALFF in the right precuneus and left middle temporal gyrus. Moreover, whole-brain exploratory FC analysis showed that the FC between the left mid-insula and parietal regions within the sensorimotor network was weaker in patients with MDD than in healthy controls. Collectively, these findings indicate that individuals with MDD display neural dysfunction mainly in the temporo-limbic circuits associated with emotion processing, the default mode network related to self-oriented information processing, and the sensorimotor network involved in sensory information processing and motor control.

Compared to healthy controls, MDD patients showed stronger brain activity in the left parahippocampus and weaker activity in the left middle temporal gyrus. The parahippocampus is located medially beneath the occipital and temporal lobes. This region serves as the primary cortical input to the hippocampus, which has important involvement in cognition and emotion (38). As a key component of the limbic system that participates in emotion regulation, the parahippocampus has been presented as an important nucleus in neurological research on MDD (17, 39, 40). Several studies have demonstrated increased activity in the hippocampus and parahippocampus in MDD (41–43). For example, Chen et al. (17) found that ALFF values in the parahippocampal gyrus were positively correlated with clinical depression scores (17). Wu et al. (16) used another resting state fMRI indicator, regional homogeneity, and reported that individuals with MDD had a higher regional homogeneity in the limbic lobe, including in the medial prefrontal cortex, insula, and parahippocampus (16). The temporal poles are responsible for evaluating emotional significance by integrating relevant contextual cues (4). Guo et al. (44) observed lower ALFF values in visual recognition circuits, including the middle temporal gyrus, in patients with treatment-resistant MDD (44). Brain activity in the temporal lobe has been found to be negatively correlated with symptom severity in patients with MDD (4). Thus, consistent with previous studies, the current study showed different ALFF patterns in regions related to emotion regulation, which indicated that MDD might be associated with impaired emotion regulation and poor integration of affective responses.

Moreover, in accordance with previous findings (26, 45–47), the present study reported that patients with MDD showed abnormal neural activity in brain regions within the default mode network. These regions—the thalamus, precuneus, and parahippocampus—are important components in the default mode network (48). The thalamus, as a hub connecting the cortex and subcortical regions, is thought to be the transformation center for all neural signals from bodily sensations projected to the cerebral cortex (49). A previous study demonstrated functional and structural abnormalities in this area in individuals with MDD (26). Alcaro et al. (43) conducted a meta-analysis and reported hyperactivity in the anterior cingulate cortex, ventromedial prefrontal cortex, and thalamic regions in patients with MDD (43). The precuneus is an important node in the default mode network that is involved in self-related information processing (50). Hypoactivity in the precuneus has been consistently observed in patients with MDD (31, 45). Liu et al. (51) found that reduced ALFF in the precuneus was associated with the onset of depressive episodes (51). The default mode network is mainly responsible for self-referential internal attention processing (52). Individuals with MDD have difficulties regulating self-oriented thoughts to generate goal-directed behaviors owing to a dysfunctional default mode network.

Surprisingly, whole-brain exploratory resting-state FC analysis in the current study revealed that individuals with MDD showed abnormal FC within sensorimotor networks, which has seldom been mentioned in previous studies. Specifically, left mid-insula–parietal FC was weaker in the MDD group than in the healthy control group. The insula has been documented as the primary region involved in interoceptive awareness (53, 54). Avery et al. (55) reported reduced activity in the dorsal mid-insula during the interoception tasks and abnormal resting-state FC between the dorsal mid-insula and limbic regions (55). This abnormal activity reflects altered information processing of interoceptive signals, which interferes with an individual’s evaluation of how external stimuli will affect their homeostatic state (56). The parietal lobe contains many sensory centers, and dysfunction in this area may result in cortical sensory abnormalities or sensory disorders (57).

Individuals with MDD show a wide variety of symptoms with respect to sensorimotor, affective, cognitive, and social function (58). Sensorimotor abnormalities in individuals with MDD manifest as psychomotor agitation and retardation. The sensorimotor network anatomically corresponds to the sensory and motor areas, including the precentral gyrus, postcentral gyrus, and supplementary motor areas. Functionally, spontaneous fluctuations in this network are likely to reflect the neural demands that support active motor behaviors (59). Northoff (58) proposed that resting-state FC within the salience and sensorimotor networks is also affected in MDD (58). Yao et al. (60) suggested that abnormal function in the sensorimotor network may result in a perceptual function deficit in MDD (60). Therefore, deficits in the sensorimotor network may be a neural cause of sensory disorders or psychomotor retardation in MDD.

In general, findings of altered activity and FC in the temporolimbic circuit (e.g., middle temporal gyrus, parahippocampus) and default mode network (e.g., precuneus, thalamus) explored in the present study were seen in individuals with MDD. It was also confirmed that these two brain structures are key brain regions involved in the neurological dysfunction seen in depression, consistent with dysfunctional emotion processing and inaccurate evaluation of self-related information observed in individuals with MDD. Furthermore, the present study found abnormal FC within the sensorimotor network in individuals with MDD, implying that individuals with MDD not only exhibit affective dysfunctional symptoms, but also have a risk of dysregulation in sensory information processing and motor control. Importantly, these findings provide new evidence for neurological research on depression.

This study has some limitations. First, the sample size was relatively small. Thus, the generalizability and interpretation of the findings are limited. Future studies should include larger samples of patients with MDD to improve the generalizability of the results. Second, the group division in this study was not sufficiently detailed. For example, the degree of neurological damage and corresponding cognitive dysfunction varies among patients with depression of different severity (16). Patients with MDD with diverse symptoms may also differ in their neurological activity (44, 45, 61). Therefore, future studies should explore the unique neural activity patterns of patients with different types of depression in detail. Third, the present study only investigated abnormal neural activity patterns of individuals with MDD using conventional resting-state activity and FC analysis. Future studies should combine advanced technical analyses with fMRI data from different modalities, such as structural fMRI and diffusion tensor imaging, to reveal the biological significance of neural activity in MDD more comprehensively.

## Conclusion

This study, using resting-state fMRI and FC analyses, found altered ALFF in the temporolimbic circuit (e.g., middle temporal gyrus and parahippocampus) and default mode network (e.g., precuneus and thalamus) in individuals with MDD. Moreover, FC between the left mid-insula and parietal regions within the sensorimotor network was weaker in individuals with MDD than in healthy controls. These results provide neural markers to identify MDD, contributing to the clinical diagnosis of affective and sensorimotor disorders. The current findings validate and extend the neurological theory of MDD, complement previous findings on cognitive neurological disorders in MDD, and further improve our understanding of the potential pathophysiology of depression.

## Data availability statement

The original contributions presented in this study are included in the article/Supplementary material, further inquiries can be directed to the corresponding author/s.

## Ethics statement

This study was approved by the Medical Ethics Committee of Zhumadian Second People’s Hospital in Henan Province (Approval no. IRB-2020-006-02). The patients/participants provided their written informed consent to participate in this study.

## Author contributions

BC and FW conceived and designed the study. XH and BM performed the data extraction and statistical analysis. HG, SL, CZ, and GW contributed to the discussion. XH, BM, and FW took the lead in writing the manuscript. All authors discussed the results and commented on the manuscript.

## Abbreviations

ALFF: amplitude of low-frequency fluctuations
FC: functional connectivity
fMRI: functional magnetic resonance imaging
MDD: major depressive disorder
ROIs: regions of interest.
