# Supplementary material for: Neural activity in adults with major depressive disorder differs from that in healthy individuals: A resting-state functional magnetic resonance imaging study
Source: Front Psychiatry. 2022 Nov 17;13:1028518. doi: 10.3389/fpsyt.2022.1028518 (PMC9712791; doi:10.3389/fpsyt.2022.1028518)
Supplement: Supplementary file 1 [file Data_Sheet_1.docx]

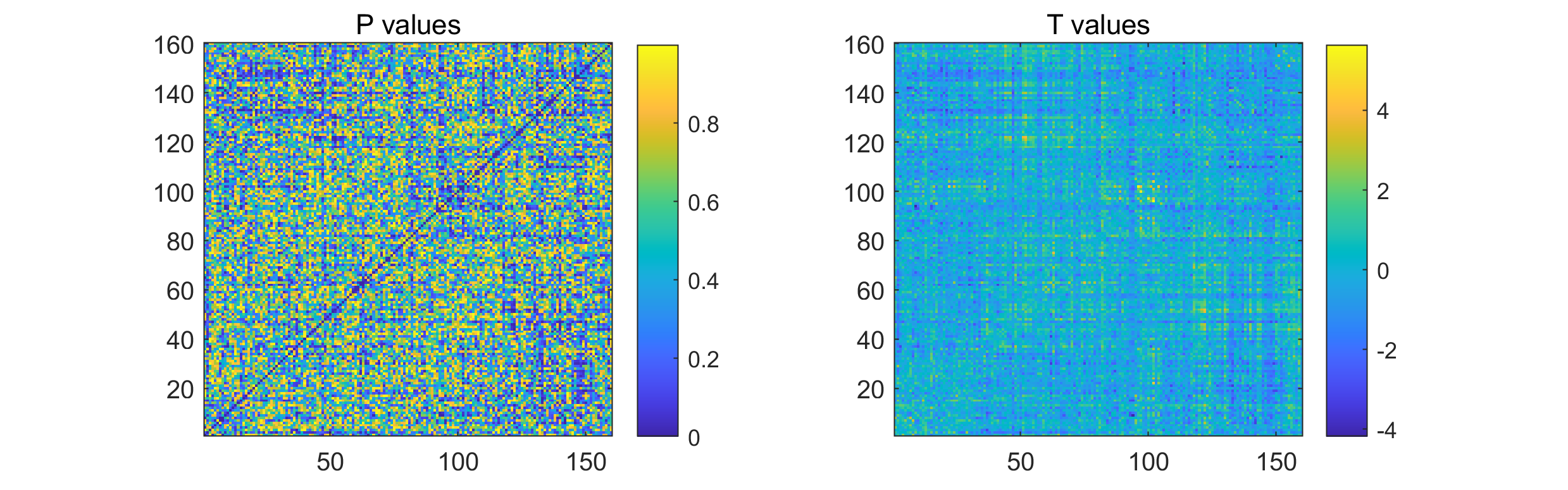


**Supplemental Figure 1.** Detailed T value and P value of two sample T test in FC corresponding to every edge
